# Feasibility of combining spatial computing and AI for mental health support in anxiety and depression

**DOI:** 10.1038/s41746-024-01011-0

**Published:** 2024-01-26

**Authors:** Brennan M. R. Spiegel, Omer Liran, Allistair Clark, Jamil S. Samaan, Carine Khalil, Robert Chernoff, Kavya Reddy, Muskaan Mehra

**Affiliations:** 1 Department of Medicine, Division of Health Services Research Virtual Medicine Program, Cedars-Sinai, Los Angeles, CA 90048 USA; 2 Department of Medicine, Division of Gastroenterology, Cedars-Sinai, Los Angeles, CA 90048 USA; 3 Department of Psychiatry and Behavioral Sciences, Cedars-Sinai, Los Angeles, CA 90048 USA

**Keywords:** Quality of life, Psychology

## Abstract

The increasing need for mental health support and a shortage of therapists have led to the development of the eXtended-reality Artificial Intelligence Assistant (XAIA). This platform combines spatial computing, virtual reality (VR), and artificial intelligence (AI) to provide immersive mental health support. Utilizing GPT-4 for AI-driven therapy, XAIA engaged participants with mild-to-moderate anxiety or depression in biophilic VR environments. Speaking with an AI therapy avatar in VR was considered acceptable, helpful, and safe, with participants observed to engage genuinely with the program. However, some still favored human interaction and identified shortcomings with using a digital VR therapist. The study provides initial evidence of the acceptability and safety of AI psychotherapy via spatial computing, warranting further research on technical enhancements and clinical impact.

The rising prevalence of mental health issues in the United States, exacerbated by factors such as the COVID-19 pandemic, economic strain, and the ubiquity of isolating technologies like social media, has outpaced the availability of psychotherapeutic care^1–4^. This situation, coupled with the high costs and stigma associated with psychotherapy, demands innovative solutions to deliver accessible, affordable, and stigma-free mental healthcare^5–7^.

Virtual reality (VR) employs spatial computing to create meaningful psychological experiences, promoting a sense of presence^8^. VR’s versatility enables users to experience serene natural settings or meditative landscapes, supporting treatments for conditions like anxiety and depression when integrated with cognitive behavioral therapy (CBT)^9^. However, personalizing CBT in VR remains a challenge, historically relying on real-time therapist interaction or pre-scripted content.

Advancements in artificial intelligence (AI), particularly Large Language Models (LLMs), provide an opportunity to enhance VR’s therapeutic potential. These models can simulate naturalistic conversations, paving the way for AI-driven digital therapists^10^. Yet, the efficacy of combining VR with AI therapy, including the establishment of a therapeutic alliance with a virtual avatar and the ability to address diverse biopsychosocial issues, has not been examined.

In this brief communication, we describe the eXtended-reality Artificial Intelligence Assistant (XAIA), a program developed at Cedars-Sinai that merges VR and AI to offer personalized, CBT-based mental health support. Users select from nine nature scenes and interact with “XAIA,” a robot avatar. Conversations are processed through a HIPAA-compliant server: audio is recorded, transcribed by speech-to-text AI, and responded to by LLM (GPT-4). The LLM output is sent through an “Appropriateness Classifier”—a stand-alone AI to detect potentially dangerous or unhelpful responses. If triggered, the LLM is again queried, and its response is again analyzed. Otherwise, the output is released to a text-to-speech AI, which finally, together with sentiment analysis meta-data controlling XAIAs expressions, plays to the user (Fig. 1). The system’s development involved iterative testing with therapists role-playing clinical scenarios, leading to continuous refinement of its psychotherapeutic communication.

**Fig. 1:**
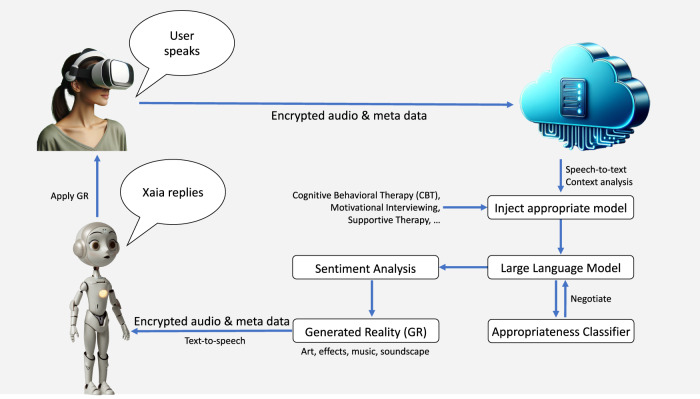
XAIA data pipeline. See text for details.

Table 1 displays characteristics of the 14 participants with mild-to-moderate anxiety and depression who interacted with XAIA, which was the number of interviews performed until achieving thematic saturation. Participants raised a wide range of biopsychosocial topics in response to XAIA’s initial inquiry, *“How can I help?”*. Table 2 presents sample participant quotes alongside XAIA’s initial response.

**Table 1:** Patient characteristics.

Total: *N* = 14				
	*n*	%	*Range*	M (SD)
Gender				
Female	6	42.9		
Male	7	50		
Non-Binary	1	7.1		
Race/Ethnicity				
White (Non-Hispanic)	6	42.86		
Black or African American	2	14.28		
Asian	0	0		
Hispanic	5	35.7		
American Indian or Alaskan Native	1	7.14		
Highest Level of Education				
8th Grade or Less	0	0		
Some High School	0	0		
High School Graduate or Equivalent	1	7.69		
Some College	6	46.15		
College Degree	3	23.07		
Advanced Graduate Degree	3	23.07		
Combined Household Income				
Less than $10,000	1	8.33		
Between $10–20,000	3	25.00		
Between $20–50,000	2	16.67		
Between $50–100,000	3	25.00		
Between $100–200,000	0	0		
More Than $200,00	1	8.33		
Prefer Not to Answer	2	16.67		
VR Experience				
No experience	5	38.46		
A little bit of experience	4	30.76		
Some experience	1	7.69		
Quite a bit of experience	2	15.38		
A lot of experience	1	7.69		
Age	14		25–73 (48)	45 (17.18)
PHQ-9	14		5–19 (14)	10.429 (4.05)
GAD-7	14		0–13 (13)	7.643 (3.52)

**Table 2:** Examples of initial topics of discussion from participant (left column) and initial response from XAIA (right column).

Initial Topic of Discussion from User	Response from XAIA
“Loneliness… Divorce, parents, spouse abuse and like substance abuse and I guess you could say partially neglect.”	“I’m sorry to hear you’ve had to experience such difficult circumstances. These situations can indeed lead to feelings of loneliness. Could you share more about your experiences with these issues and how they’ve been affecting your thoughts and feelings?”
“Yeah, my last relationship with my girlfriend, I believed, was unresolved. And since then I’ve been having night sweats.”	“It sounds like the unresolved nature of your last relationship might be affecting your emotional well-being. Could you share more about what made the relationship feel unresolved and how it has impacted you?”
“Stress. Moving, financial, my mother passed away. I can’t sleep.”	“I’m really sorry to hear about your mother’s passing and the stress you’re going through right now. The mix of grief, moving, and financial worries can indeed be overwhelming. Remember, it’s okay to feel this way. How long ago did your mother pass? This can help me understand your grieving process a bit better.”
“I’m just having anxiety about like a lot of pain that I’m feeling.”	“I’m sorry to hear that you’re feeling this way. Experiencing pain and frequent discomfort can indeed trigger feelings of anxiety. Could you talk a bit more about how it’s been affecting your everyday life emotionally?
“I need some help dealing with the fear of loss, particularly the fear of loss of my wife.”	“I’m sorry to hear that you’re experiencing this fear. Can you share more about the specific situations or events that have led to this fear of losing your wife?”
“I’m dealing with frustration and I’m dealing with what I feel are not good people and I wish that I was better so that I can go home and also live a more normal life without having to keep coming back to the hospital.”	“I see, it sounds like you’re going through a tough time. Hospital stays can indeed be challenging and can trigger feelings of frustration. Coping with such situations often brings out a wide range of emotions. Could you tell me more about what makes you feel that you’re dealing with not good people?”
“I’ve just recently got laid off.”	“I’m really sorry to hear about your job loss. It must be a challenging time for you. Could you please provide more details about the situation surrounding your layoff and how it has affected you?”

The digital avatar was observed to apply essential psychotherapeutic techniques. It frequently made observations that reflected an understanding of the user’s issues (e.g., “Your worries about your health and your living situation are clearly causing you a lot of distress”) (Supplementary Table 1). By normalizing feelings (“It’s understandable to feel like a failure when life gets overwhelming”), expressing sympathy (“I’m really sorry to hear about your job loss”), and showing empathy (“It must be a challenging time for you”), XAIA was noted to communicate understanding and compassion. It also offered validation and praise where appropriate (e.g., “Your practice of not taking these relationships for granted is truly commendable”). This combination of therapy techniques, such as restatement, empathy, and probing inquiry, can be seen in this example:

**Participant:** I was left out of her will because I followed my dreams. I’ve never really figured out how to deal with that. I just move forward as best I can.

**XAIA:** I’m sorry to hear that you felt rejected in such a definitive way, especially when you were pursuing what’s important to you. It must have been a tough experience. What thoughts tend to come up when you think about this situation?

We also observed situations where XAIA’s application of psychotherapy was sub-optimal. In some instances, XAIA excessively questioned participants, reminiscent of less experienced therapists. For example, when a participant mentioned job loss, XAIA focused more on coping strategies than on exploring the individual’s emotional response and the deeper impact of the event. In cases of serious life challenges, such as a cancer diagnosis or relationship issues, XAIA repeatedly inquired about coping mechanisms, diverting from a deeper emotional exploration.

XAIA’s approach to introducing CBT techniques like mindfulness and cognitive restructuring sometimes lacked clarity, often mentioning these methods without sufficient explanation or contextual relevance. This contrasts with the more gradual and informative approach of experienced therapists, who ensure patient comprehension before advancing.

No participants stopped therapy due to distress and VR-induced vertigo was not reported. One individual ended a session early because of neck discomfort. XAIA encouraged seeking professional advice when appropriate, as seen when one user’s gastrointestinal complaints led to XAIA advising a medical consultation.

In simulations of critical scenarios, like domestic violence, XAIA provided resource recommendations and internet safety tips, such as using “incognito mode” so an abusive partner could not monitor online activity. For simulated suicidal thoughts, XAIA directed users to a suicide prevention hotline, emphasizing its own limitations in emergency response.

Participants highlighted potential advantages of XAIA over traditional therapy, noting its appeal for those who are “less open” to in-person sessions or seek anonymity (Supplementary Fig. 2). XAIA was described as beneficial for the lonely, homebound, or those in remote areas without access to specialists. A nonjudgmental nature was noted, with a participant remarking, “I felt less judged and accepted.”

Participants reported a heightened sense of relaxation within the VR nature scenes, with many expressing a preference for the calming visuals and spatial audio that enhanced their engagement. Feedback for improvements included adding varied sceneries and integrating more dynamic elements, like light and water movement, to enrich the immersive experience further.

While XAIA was favored by some for its convenience, others valued the deeper engagement with human therapists, noting the accountability a real therapist enforces and their ability to uncover underlying issues. Some participants found it odd to connect emotionally with AI. Participants suggested enhancements like voice options, screen positioning, session timers, and the ability to pre-share personal beliefs to make interactions with XAIA more personalized and effective in creating a therapeutic alliance.

The Supplementary Information provides further results, including evidence of achieving thematic saturation and other perceptions about interacting with an AI therapist via spatial computing.

In summary, this qualitative study evaluates a VR/AI program designed to simulate a human therapist using GPT-4, across a socioeconomically diverse patient group with mild-to-moderate anxiety or depression. Participants described the digital avatar as empathic, understanding, and conducive to a therapeutic alliance. While immersive VR environments fostered a calming space for personal reflection and emotional sharing, some participants expressed a preference for the nuanced understanding and accountability provided by a human therapist.

Future research should investigate longitudinal effects on clinical outcomes and modify the program’s AI to further enhance therapeutic communication styles. This study identifies the potential of combing spatial computing and AI as a supplemental resource in mental healthcare, offering an additional approach to support the growing need for accessible mental health interventions.

## Methods

### Development of XAIA

XAIA was developed with the goal of offering AI-enabled, self-administered, mental health support within VR environments. Upon launching the program, users choose among nine immersive nature scenes including water-based, terrestrial or celestial environments. Within each scene, the user is greeted by a robot named “XAIA” who represents the therapist.

**Fig. 2:**
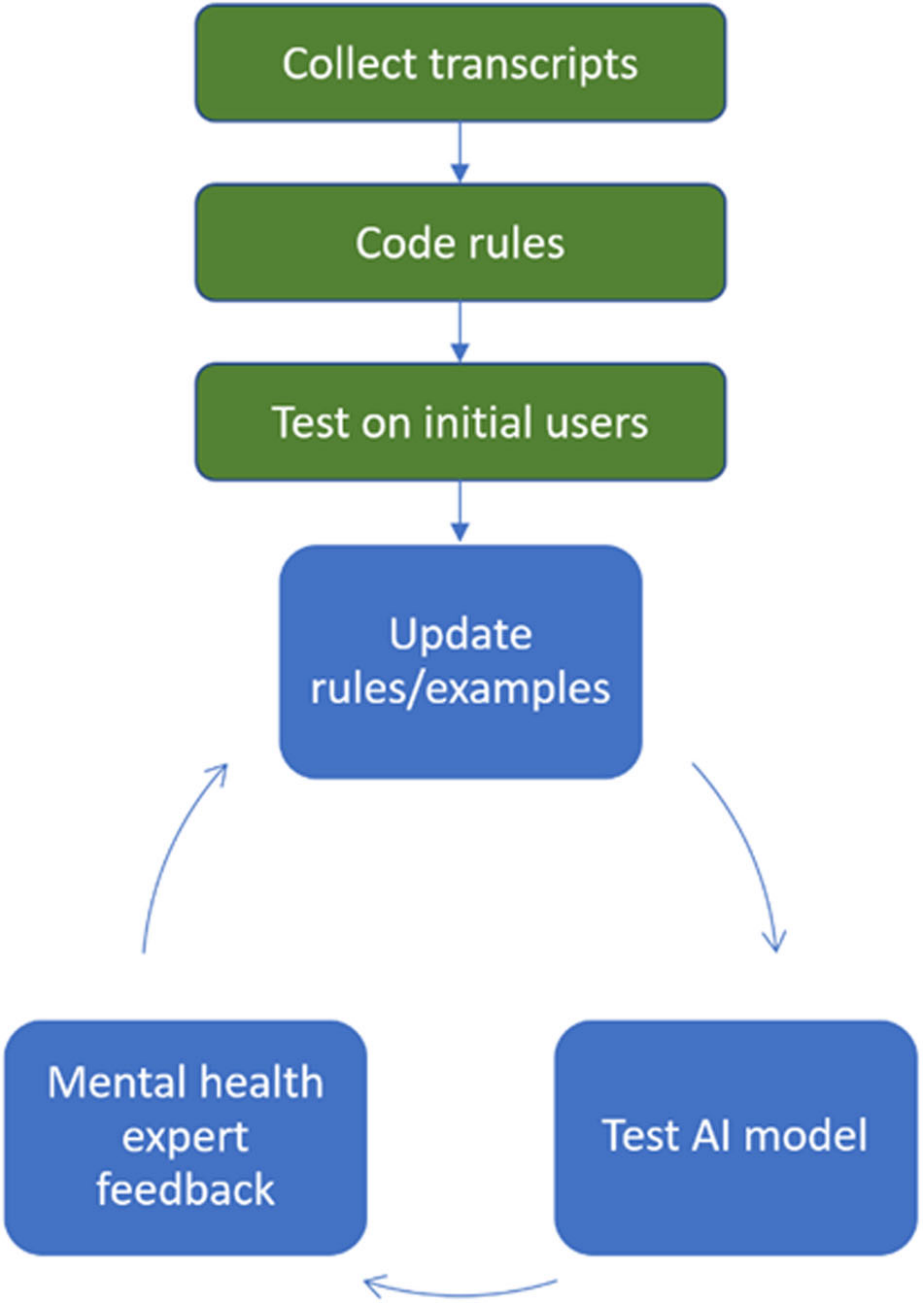
Iterative process for developing XAIA system prompts. See text for details.

During initial testing with GPT-4, we noted that its responses were inconsistent with psychotherapy best practices. For example, the LLM was too quick to offer advice and did not expend time establishing rapport. Thus, we employed a structured protocol with the goal of attempting to train GPT-4 to respond in a manner akin to a human therapist (Fig. 2).

Initially, we collected transcriptions of CBT patient-therapist interactions performed by an expert psychotherapist to improve the program’s adherence to the style and cadence of an experienced human therapist. From these, we discerned recurring exchanges and encoded these patterns into GPT-4’s system prompts. For instance, a rule was established: “Show empathy and understanding to validate [first name]’s feelings.” Accompanying this rule were exemplar responses, such as: “It must be challenging facing such circumstances” and “I understand how this has led to your feelings of [emotion].” Similarly, we added system prompts to reframe cognitive distortions, identify automatic negative thoughts, approach conversations without judgment, and avoid technical jargon or patronizing language, among a list of over seventy other psychotherapy best practices.

XAIA was programmed to identify the degree to which users were engaging fully, and to re-evaluate its line of inquiry if the system detected waning engagement. XAIA was also prompted to use the VR environment in a therapeutic manner, for example introducing an interactive breathing exercise within the virtual space if determined appropriate by the AI.

If at any time the user expressed hints of suicidal ideation, then they were directed to seek crisis intervention and immediate support and were provided with information for emergency services. If the user raised medical issues outside the scope of talk therapy, XAIA was programmed to advise the user to seek care from a medical healthcare professional.

Working with an expert psychotherapist and an experienced psychiatrist, we iteratively updated and refined the system prompts to optimize idealized responses of a compassionate, non-judgmental, and helpful therapist. The system was then systematically evaluated by licensed mental health professionals assuming the roles of patients across a wide range of clinical scenarios (e.g., discussing anxiety, depression, work-life balance, relationship issues, trust issues, post-traumatic stress, grief, self-compassion, emotional regulation, and social isolation, among other reasons people seek talk therapy). Their detailed feedback allowed further refinement and expansion of the system prompts and model responses.

Subsequently, we broadened our user base, enabling interactions with XAIA within a supervised environment. Each transcript was reviewed by a mental health expert, pinpointing areas of potential improvement. This iterative process, comprising prompt adjustment followed by evaluation and expert review, was repeated over a hundred times. We continued this cycle until feedback consistently indicated substantial improvement, with critiques becoming increasingly specific and infrequent.

### Study participants

We sought to recruit up to 20 adult participants with the goal of ending recruitment once achieving thematic saturation. We recruited via IRB-approved social media posts and direct recruitment from clinicians.

Participants were required to speak English and obtain a score on the Patient-Health Questionnaire between 5 and 19 or a score on the Generalized Anxiety Disorder 7-Item Scale between 5 and 14, representing mild-to-moderate depression or anxiety, respectively. We excluded individuals with motion sickness, facial or head deformities, seizure in the past year, pregnancy, or being legally deaf or blind. The study protocol was approved by the Cedars-Sinai Medical Center Institutional Review Board (IRB STUDY00002753) and rated by the IRB as minimal risk and requiring verbal consent. Our study was conducted in accordance with the International Conference of Harmonization Guidelines for Good Clinical Practice and the Declaration of Helsinki.

Consenting patients visited Cedars-Sinai to participate in a single therapy session. During their visit, participants were briefed on use of the headset (Meta Quest 2) and then engaged privately with XAIA for up to 30-min. A licensed mental health professional remained available if needed. Participants then engaged in a de-briefing interview led by a qualitative researcher.

### Qualitative analysis

Two qualitative researchers lead inductive thematic analyses to derive themes from the transcribed interviews. They created codes and labels from the unstructured data through iterative passes, with each subsequent pass refining and aggregating the codes into themes supported by direct quotes. This process occurred after each visit, and continued until no new codes were identified, thus indicating thematic saturation. We quantitatively tracked saturation by graphing the emergence rate of new second-level pattern codes by interview, generating a cumulative count of novel second-level codes over time (Supplementary Fig. 1). The Supplementary Information provides further details regarding the qualitative analyses.

All original text was written by the authors for a full publication. After editorial guidance to format the paper for a brief communication, we employed Chat GPT-4 to help modify the text so it fit within the compressed format.

### Supplementary information

Supplementary Information

## Data Availability

The Supplementary Information provides a sample anonymized transcript shared with patient permission. Additional patient transcripts are not publicly available as they contain information that could compromise research participant consent.

## Code availability

No unique code was written for this study. We used the pre-existing GPT-4 software along with a prompting strategy as described in the methods.
