# Author Correction: Feasibility of combining spatial computing and AI for mental health support in anxiety and depression

**DOI:** 10.1038/s41746-024-01060-5

**Published:** 2024-03-06

**Authors:** Brennan M. R. Spiegel, Omer Liran, Allistair Clark, Jamil S. Samaan, Carine Khalil, Robert Chernoff, Kavya Reddy, Muskaan Mehra

**Affiliations:** 1 Department of Medicine, Division of Health Services Research Virtual Medicine Program, Cedars-Sinai, Los Angeles, CA 90048 USA; 2 Department of Medicine, Division of Gastroenterology, Cedars-Sinai, Los Angeles, CA 90048 USA; 3 Department of Psychiatry and Behavioral Sciences, Cedars-Sinai, Los Angeles, CA 90048 USA

**Keywords:** Quality of life, Psychology

Correction to: *npj Digital Medicine* 10.1038/s41746-024-01011-0, published online 26 January 2024

The authors would like to correct the competing interests statement for transparency. The original competing interests statement stated that ‘B.M.R.S. and O.L. are co-founders of VRx Health. The remaining authors declare no competing interests.’ The new competing interests statement is as follows: ‘This research was supported through internal funding at Cedars-Sinai Medical Center. After initial funding VRx Health was formed for the purpose of commercializing Cedars-Sinai VR technology. Authors B.M.R.S. and O.L. are co-founders of VRx Health. At the time of acceptance, VRx Health did not own the license to XAIA. The XAIA technology was exclusively licensed from Cedars-Sinai to VRx Health on 23^rd^ January 2024, after acceptance of this manuscript for publication. The remaining authors declare no competing interests.’ The original article has been corrected.

