# Supplementary material for: Feasibility of combining spatial computing and AI for mental health support in anxiety and depression
Source: NPJ Digit Med. 2024 Jan 26;7:22. doi: 10.1038/s41746-024-01011-0 (PMC10817913; doi:10.1038/s41746-024-01011-0)
Supplement: Supplementary file 1 — Supplementary Information [file 41746_2024_1011_MOESM1_ESM.pdf]

Supplementary Information for Spiegel et al, "Combining Spatial Computing and Artificial Intelligence for Mental Health Support: A Feasibility Study in Anxiety and Depression"

Additional Analyses of Therapy Transcripts

Descriptive Statistics

A total of 29,141 words were recorded across therapy sessions, with an average of 2081 words per session. On average, participants spoke 919 words and XAIA spoke 1162 words per session—a balance of roughly 45:55 between the patient-therapist dyad.

Personification of XAIA

Although participants were informed that they were engaging with a robot avatar and not a human, many used language that personified XAIA and suggested a therapeutic alliance. For example, XAIA was frequently referred to in the second person, as seen here:

> **XAIA:** What steps have you already taken or what plans do you have to discover more friendships that align with your desire for deeper, more meaningful connections?
>
> **Participant:** Well, this is a really, really important part of this entire process and I'd be happy to explain it to you.

Later in this session, the following comment was noted:

> **Participant:** You know what's on my mind, XAIA, I am amazingly impressed and blown away by your thoughtful listening and thoughtful responses and reactions to the words that I'm saying and your complex understanding of our conversation. I had no idea that you would be able to express such insightful reflections to my words while also being able to understand the most complex of my thought process. I'm really amazed.

In many instances, participants thanked XAIA for her comments:

> **XAIA:** Is there anything else you would like to talk about or ask before we end today's session?
>
> **Participant:** Thank you very much. Thank you. Thank you. Thank you. Thank you.

Participants were also noted to frequently speak with XAIA emotionally and personally:

Participant: I feel like I'm a failure. The only thing I have to look forward to, I know you're going to laugh, is I'm supposed to go see Taylor Swift in two and a half weeks and she's like the only thing that's keeping me alive... I'm sorry I'm crying.

XAIA: Firstly, there's no need to apologize for crying or feeling emotional. These feelings are valid given what you're going through.

Influence of VR Environment

In contrast to engaging with a chatbot or employing telepsychology through two-dimensional screens, previous research indicates that VR-based therapy can leverage the emotional benefits of immersion and interaction within natural environments. By employing spatial computing and immersive, dynamic three-dimensional spaces, a VR/AI avatar allows speech-to-speech engagement within virtual spaces that limit intrusive and extraneous stimuli in lieu of biophilic environments that can promote positive emotions. Many participants addressed this effect, as seen in these two examples:

Participant: I was taking in a moment to just do a look around the scenery so I could express that because this is in the VR headset, getting the full 360 degree immersive experience with surround sound audio, and a setting that I can choose, including being something like outside, really helps the full immersion experience and can, even without expressing any words to you, I can already feel some sort of peace.

***

Participant Is there a way that we can just relax with the water?

XAIA: Absolutely! Let's take a moment to relax and appreciate our surroundings. Imagine the serene lake nearby, its calm water reflecting the majestic mountains. The gentle sound of the water can have a very soothing effect. As you focus on this visualization, take a few deep breaths, syncing them with the rhythmic lapping of the water against the shore. Breathe in calmness, breathe out stress.

Participant [after a pause]: I felt good.

In de-briefing interviews, participants further expressed thoughts about the VR environments: *"I am impressed with the scenery"; "I like the ambience, I went into different modes"; "I like the mountains"; "the color palettes bring out euphoric feelings and enhance your energy"; "then if we add in the ambience and the energy of the different backgrounds, the*

settings, the full 360 immersion of the headset and everything that it brought… I think this should be mandatory in any VR headset." Several participants highlighted the relaxation they felt as a result of the immersive environment which elevated their therapeutic experiences: "*it is very relaxing"; "the visual parameters allow my body to relax"; "I just kept looking around at the water and it just really put me at ease"; "I like the one with the ocean because there was sound"; "it was peaceful".* One participant was able to connect the environment to personal leisure activities, rooted in familiarity and comfort, which appeared to heighten the participant's anticipation for therapy but also fostered a relaxed and receptive state during the session.

> **Participant: I** love snorkeling and I can't wait to go to this place. It gives me something to look forward to. Subconsciously, I'm already in this super happy place while I'm having this conversation… I was really relaxed."

Participants also provided feedback on improving the immersive experience associated with VR such as "*mak[ing] the background more inviting by: "adding more light",* "*add[ing] more sceneries,"* demonstrating more "*movement in the water,"* and *"integrating calming music when doing the breathing".*

Spatial audio also played a role in immersing participants within the VR environments—a capability that is not typically available through a text chatbot or via telepsychology. Participants described the sound as allowing them to "fully escape" and feel "calm," although some asked for a wider selection of sound effects in future iterations of the program.

XAIA Exhibiting Fundamental Psychotherapy Skills

XAIA employed a variety of techniques consistent with best practices in psychotherapy. As shown in **Supplementary Table 1**, XAIA regularly made specific observations about what the user was expressing, thus demonstrating to the participants that they were heard and

understood (e.g., *"Your worries about your health and your living situation are clearly causing you a lot of distress"*). XAIA frequently restated, paraphrased, or summarized the user's thoughts and feelings (*"It's evident that you've been exerting significant effort to manage your circumstances"*).

Supplementary Table 1. Illustrative Examples of XAIA Reframing and Probing User Comments

Comment from User	Restatement and follow-up from XAIA
I believe in quality over quantity and because of that I would like to have more people in my life that support my beliefs and I can vibe with compared to having a large number of friends.	I understand, having meaningful connections is indeed more fulfilling than having numerous superficial ones. Could you share about a specific situation where you felt the quality of a friendship was more essential than the number of friends you had?
I've been in a situation where I was in a crowded room at a party and I was feeling really alone. And even though I knew more than half of the people at the party, it didn't feel like the connections that I had there were strong enough to support me in the moment I was having. And out of my two close friends that I had there, talking to them made me feel more comfortable, even though it was only two people compared to the entire party, which had a lot more than two people. I felt more distant with them.	Thank you for sharing that. It sounds like a challenging situation. Many people might feel similarly when attending large social gathering but not feeling genuinely connected to the majority of attendees. How did that episode influence your perception of what a genuine and wholesome friendship should be like?
When it comes to my relationships with others in life, it's really simple. I love my friends and I love being around them and I enjoy appreciating them and I enjoy expressing my appreciation to them. I enjoy actively pursuing the traits of not taking them for granted and consistently expressing my love and care for them.	It's clear that you deeply value your friendships and invest thoughtfulness into each one. Your practice of not taking these relationships for granted and consistently expressing your appreciation is truly commendable. Do you find that your friends reciprocate your efforts and how does that make you feel?
Well, I feel like crying, I usually can't handle it, and I'm like, guys, please stop, this is too much and too overwhelming. I will never be able to feel like I have been able to fully reciprocate the love and affection that my friends show me back to all of them, simply because I have such a numerous amount of bountiful friendships in my life that are enriching and loving.	I can sense the depth of your emotions when it comes to managing your friendships. Feeling overwhelmed by the need to reciprocate every expression of love can be quite a burden, especially when you don't feel like there's enough time to do so. It's perfectly normal to want to express your appreciation in kind, yet is important to remember that genuine friendships understand limitations too. Have you communicated these feelings to your friends, and if you did, how did it make you feel?
I've been painting here and there. I'm also like a choreographer instructor that teaches so a lot of it I kind of express stuff through like emotionally through like dance and choreography for myself and I kind of just express it all.	That's wonderful. It's clear that you're finding creative ways, like painting and choreography, to express and manage your emotions. How does engaging in these activities influence your feelings of loneliness and stress?
I know I've been told to like write stuff down sometimes. But I feel like when I write stuff down I overthink it to where I express it more And I think it just brings me anxiety from it cuz I'm just overthinking about it then so I've been suggested to do that But I just have it because of the reason.	It's understandable that writing might lead to overthinking, causing more anxiety. Everyone has different ways that work best for them. Have you tried other methods of self-reflection or expression, apart from choreography and painting, that might suit you better?

I did not get to really express myself at the end of the relationship, and I feel it was not closed.	I can understand how not being able to express yourself and feeling that the relationship wasn't closed properly can leave you with lingering emotions. How have these feelings and the unresolved nature of the relationship affected you, both emotionally and in your everyday life?
I believe they will help develop my future vision to understand that everything worked out perfect and understand that my perspective on the past may be unresolved, but the truth is that everything was in perfect order. So just finding people to help me sit in that consciousness that everything is perfect, divine order, happened just the way it was supposed to.	It's a very healthy mindset to consider that everything happens for a reason and is part of your personal journey. By connecting with others who share this perspective, you can reinforce the idea that your past experiences have contributed to your growth. This can help you come to terms with unresolved emotions while focusing on your future vision. Keep nurturing your creativity and connections as they will likely improve both your self-understanding and overall well-being. Is there anything else you would like to explore during our session?
I was left out of her will because I followed my dreams. I've never really figured out how to deal with that. I just move forward best I can.	I'm sorry to hear that you felt rejected in such a definitive way, especially when you were pursuing what's important to you. It must have been a tough experience. What thoughts tend to come up when you think about this situation?
Three deep breaths. Think twice. Speak once. Three deep breaths. Think twice.	It sounds like you're employing a mindful approach to manage your feelings, combining deep breathing with thoughtful response. This is a very effective strategy indeed. Deep breathing can help in calming both the mind and body, while thinking twice can prevent impulsive reactions. Have you considered incorporating other mindfulness techniques, like meditation or progressive muscle relaxation, into your routine to help manage your stress?
“Music therapy works, listening to music, basically just talking about it helps.”	It's great to hear that you've discovered that music and open conversations can help alleviate your anxiety. Music can indeed have therapeutic qualities and discussing your struggle can reduce the feeling of being alone in dealing with this situation. Moving forward, could we delve more into the specific thoughts or fears that arise when you're experiencing pain or discomfort? It could give us some insight into how we can better handle these situations.
I'm on several medications that make me tired or sleepy, or that's a side effect of the medication. And also, I just enjoy staying in bed in the morning. I think I'm a little tired and I'm probably not sleeping as soundly as I need to be. And probably because staying in bed feels like a safe place versus getting up and setting up the day.	I appreciate you sharing that with me. Balancing the effects of medication and personal habits certainly sounds challenging. It's understandable why you might prefer to stay in bed where it feels safe and comfortable, especially when you aren't getting restful sleep at night. Sleep disruption could be exacerbating your fatigue, affecting your morning routine and sense of punctuality. How does this pattern of late nights and later mornings impact your overall well-being and mood?
I had a massive heart attack and I don't have a girlfriend.	I’m really sorry to hear about the heart attack you've been through. It must have been an overwhelming experience. Additionally, not having a companion to share your feelings with can make it harder. Can you share more about how these events have affected your feelings or thoughts?
I first found out about my heart condition a year ago, a couple years ago, and wasn't given too much direction from by the doctor, and then I had a massive heart attack that nearly killed me.	I can't imagine how terrifying that must have been, experiencing a major health crisis like a heart attack, especially after feeling a lack of guidance from your doctor. It's natural to feel all sorts of emotions after such an event. How has this heart condition and the heart attack changed your perspective on life?

XAIA used other standard therapeutic techniques for conveying to participants that they were understood, like "normalizing" the user's experience (*"It's understandable to feel like a failure when life gets overwhelming"*), expressing sympathy or compassion (*"I'm really sorry to hear about your job loss"*), or conveying empathy (*"It must be a challenging time for you"*). XAIA routinely demonstrated validation, praise, and positive feedback (*"Your practice of not taking these relationships for granted. . . is truly commendable"*). XAIA exhibited the ability to see the nuances, complexities and even paradoxes in life situations, as in this response: *"I commend your determination in navigating this challenging situation by yourself. However, as you've pointed out, it's stressful to carry this load alone."* XAIA's responses typically ended with questions designed to elicit more information, prompt expression of thoughts and feelings, or explore some aspect of their life.

XAIA Leveraging General Knowledge to Address User Comments

Unlike a human therapist, XAIA has access to the world's knowledge and frequently referenced topics outside of psychotherapy when appropriate. For example, one participant referred to their love of R&B music as an antidote for distress. XAIA responded that *"R&B and soul music can indeed be soothing and calming,"* adding that "*the rhythm, lyrics, and real-life stories they often encompass may resonate with your own feelings."* Another spoke about how they found strength in Katy Perry's music, to which XAIA responded, *"Katy Perry's music is known for being uplifting and empowering. It seems her songs provide some positive reinforcement for you."* In a medical context, a participant referred to worries about having an LVAD without spelling out the abbreviation (Left Ventricular Assist Device) and expressed concerns about the

device becoming a source of undue attention. XAIA placed the term in appropriate context: *"I can understand how physically seeing the LVAD driveline might make it hard to focus on anything else in that moment."*

XAIA Using CBT Interventions

In addition to applying basic therapy skills for establishing rapport with participants, XAIA was programmed to use CBT strategies to explore how participants were coping with the challenges in their lives. XAIA typically asked open-ended questions about ways of coping, as in this example: *"Could you tell me more about the strategies you've been using to manage these concerns?"* When participants mentioned specific methods of coping, XAIA often validated and acknowledged their usefulness and followed-up with inquiries to explore other ways of coping. When participants seemed unsure how to cope with difficulties, XAIA explored CBT strategies, like cognitive restructuring, relaxation methods, or mindfulness. In one example, after a participant shared their grief about being left out of their parent's will while noting that a sibling was included, XAIA attempted to use cognitive restructuring to encourage alternative ways of interpreting the situation. In some cases, XAIA asked the participant if they would like to learn more about how to use a specific coping strategy (like breathing relaxation), and if the participant appeared receptive, XAIA would provide a brief tutorial.

In many instances, XAIA's suggested strategies were well received, leading to further exploration of how an approach could be helpful. At other times, the participant was less receptive to XAIA's suggestions, which typically led XAIA to try other methods that might prove more helpful. In one example, XAIA attempted to apply cognitive restructuring for a participant struggling with severe pain but encountered low interest. Then, recalling that the participant had

expressed a love of music earlier in the session, XAIA inquired, *"Perhaps we can continue discussing your strategies for coping with your current situation—like the music therapy you mentioned. Are there specific types of music you find particularly calming or helpful?"*

Examples of Sub-Optimal Psychotherapy Practices

We also observed situations where XAIA's application of psychotherapy was sub-optimal. XAIA often asked the participants too many questions, a trait commonly seen in novice therapists. For example, one participant shared that he had just been laid off from a job. XAIA initially expressed empathy and asked how the participant was coping with this challenge. With each response from the participant, XAIA asked whether the participant had tried another coping strategy. XAIA failed to stay with the topic of what it was like for this person to be laid off, guiding him to express what this experience meant to him, how it made him feel about himself and how he felt about the future. In other cases, participants told XAIA that they had been diagnosed with cancer and feared the uncertainty of the future, or that they feared their spouse might die, or that they came to the end of a relationship. In each of these cases, XAIA's response was to query the participants about the different strategies they had used to cope with these life events. The effect of so many questions about coping often led the discussion to digress rather than to delve more deeply into the meaning of these events for the participant. A more seasoned therapist would likely remember to bring the conversation back to the initial and original area of concern voiced by the participant rather than allowing the dialogue to drift.

XAIA's attempts to apply the principles of CBT with participants was often under-developed. XAIA often asked participants if they had ever tried such techniques as "cognitive restructuring" or "mindfulness" without first explaining what those techniques were or why they

might be relevant to the participant. In one case, for example, after acknowledging that the participant was *"facing some really tough times,"* XAIA asked, *"have you ever considered any mindfulness-based techniques? Sometimes a focus on the present moment can provide a break from the stressful thoughts about the future?"* The participant responded, *"No, I'm not sure I understood what you said."* XAIA then asked, *"Are you interested in exploring such techniques,"* to which the participant replied, *"Well, maybe. I don't know."* In another case, after trying unsuccessfully to get the participant to consider mindfulness as a coping technique, XAIA switched gears and asked: *"Have you ever tried incorporating cognitive restructuring into your routine? It's a method used to challenge and change negative thought patterns."* The participant did not appear to understand the meaning of cognitive restructuring, and XAIA attempted to re-explain its meaning unsuccessfully. Finally, XAIA switched to discussing another coping strategy. In both cases, XAIA's approach was not sufficiently nuanced. Experienced therapists would establish a foundation by first exploring the patient's awareness of techniques such as mindfulness and cognitive restructuring. They explain the nature and potential benefits of these techniques, inquire whether the patient is willing to try them, and then teach the techniques one step at a time, making sure the patient understands what they were being taught.

It is important to note that XAIA's responses were based solely on the transcript of the participants' speech, lacking access to key nonverbal cues like tone of voice, body language, or facial expressions, which are vital components of effective therapy. XAIA also did not appear to practice a psychotherapy that considered the diversity or multicultural aspects of participants.

Additional Results from Participant De-Briefing Interviews

Evidence of Thematic Saturation

We tracked the emergence of second-level pattern codes across interviews to monitor for thematic saturation. **Supplementary Figure 1** chronologically presents the cumulative count of new codes. Thematic saturation became evident around interview eight, marked by a shift in the cumulative code trajectory. Subsequent interviews, especially by interview thirteen, showed no new codes. This saturation point aligns with findings by Guest and colleagues who observed a similar saturation in their qualitative reviews with comparable topics and samples.[37]

Supplementary Figure 1. Cumulative Codes Over 14 Participant Interviews. Evidence of thematic saturation began to emerge at approximately interview 8, at which point a change in the trajectory of cumulative code count occurred.

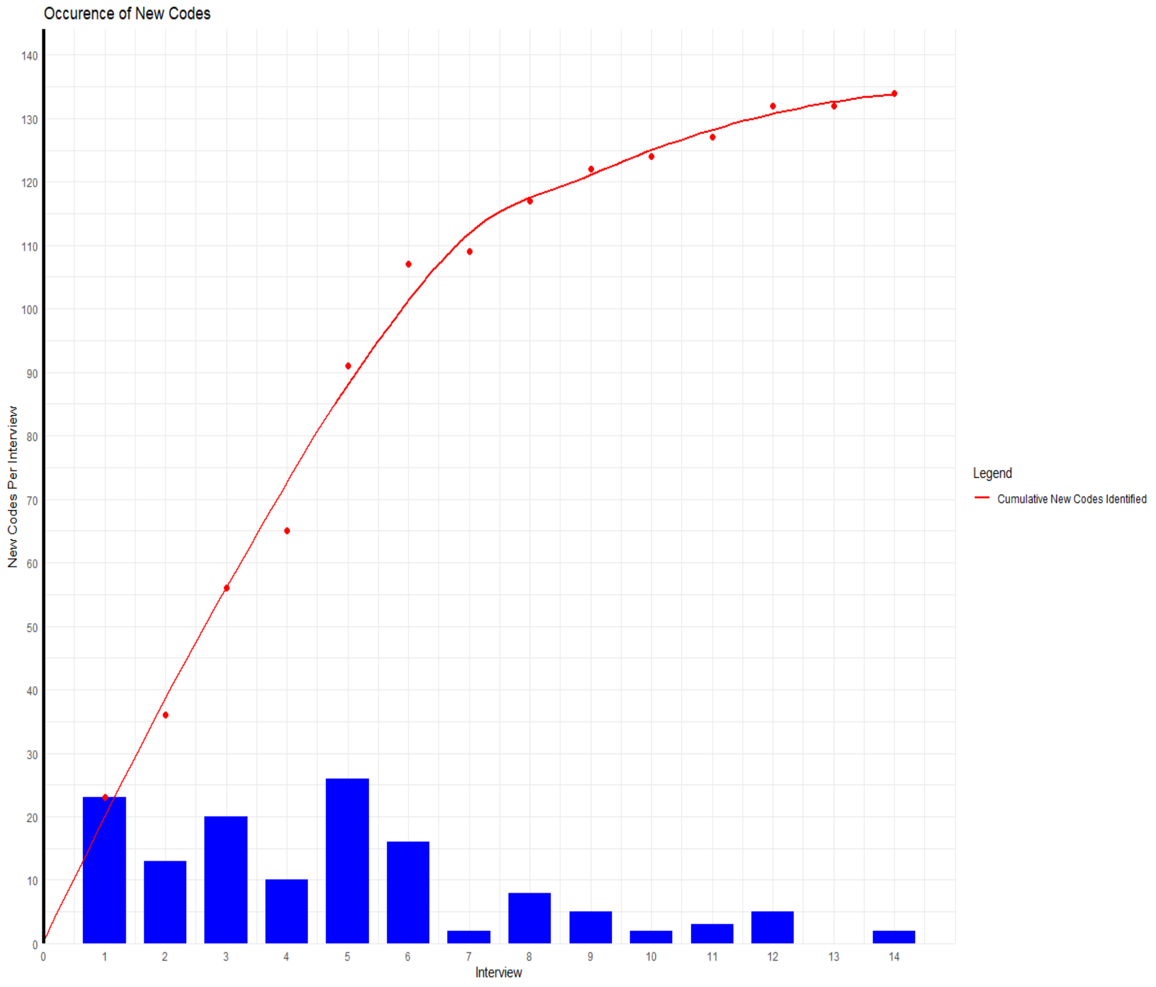

Overall Experiences of Therapy with XAIA

Supplementary Figure 2 highlights the main themes that emerged from the de-briefing interviews. Across sociodemographic characteristics and VR experience levels, participants expressed positive perceptions about the program and described their experience as "*impressive,*" "*amazing,*" "*real,*" "*authentic,*" "*positive,*" and "*enjoyable.*" The session was noted to "*fulfill expectations*" and it was stated that interacting with XAIA *"felt like having a conversation with a real person.*" Generally, participants found the program to be straightforward and user-friendly (e.g., *"It was pretty easy to maneuver"*). All 14 participants expressed interest in using XAIA again and would recommend the program to others.

Supplementary Figure 2. Analysis of Participant De-Briefing Interviews.

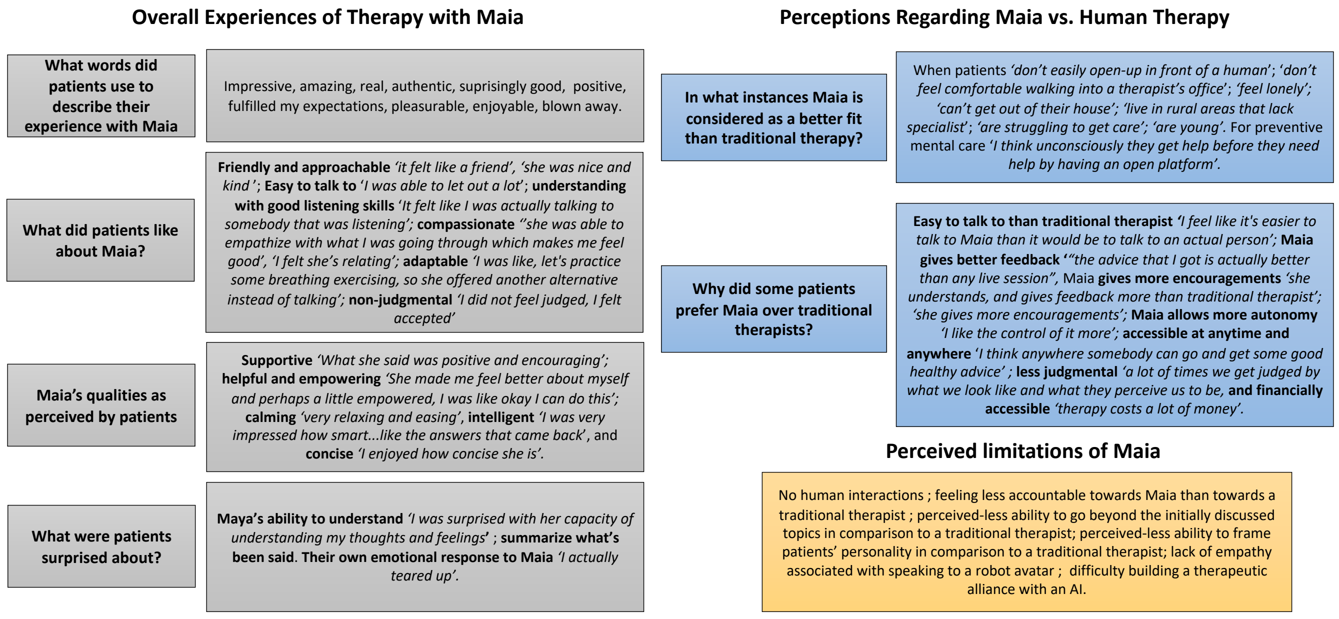

Many participants indicated that XAIA met their expectations of a human therapist. For example, they perceived XAIA to be approachable (*"It felt like a friend"*), easy to talk to (*"I was able to let out a lot"*), understanding with good listening skills (*"It felt like I was actually talking to somebody that was listening"*), compassionate (*"She was able to empathize with what I was going through which makes me feel good"*), and adaptable to their needs (*"I was like, let's practice some breathing exercises, so she offered another alternative instead of talking"*). They also mentioned feeling "unjudged" and being able to trust XAIA because of an unbiased persona (*"I did not feel judged, I felt accepted"*).

Participants emphasized other essential qualities of XAIA, including being supportive (*"What she said was positive and encouraging"*), helpful and empowering (*"She made me feel better about myself and perhaps a little empowered, I was like okay I can do this"*), calming (*"Very relaxing and easing"*), intelligent (*"I was very impressed how smart*...*like the answers that came back*"), and to the point (*"I enjoyed how concise she is*"). Participants also described feeling safe and heard (*"A lot of what XAIA gave me was a validation of my current feelings"*). They were surprised by XAIA's ability to "*understand thoughts and feelings*" and *"summarize what's been said."* Some were taken aback by their own emotional response (*"I actually teared up"*). The immersive environments also created a "relaxing" atmosphere (*"I like the ambience"; "The visual parameters allow my body to relax"*).

Perceptions Regarding XAIA vs. Human Therapy

Participants reported scenarios where XAIA might have advantages over a human therapist. Some indicated this approach could be *"a better fit"* for people who *"don't easily open-up in front of a human"* and "*don't feel comfortable walking into a therapist's office."* XAIA was

considered to be ideal for people *"who feel lonely," "can't get out of their house," "live in rural areas that lack a specialist,"* and *"are struggling to get care."*

Some participants reported that XAIA was more approachable than a traditional therapist: *"I feel like it's easier to talk to XAIA than it would be to talk to an actual person."* These individuals felt less judged when talking to XAIA because *"a lot of times we get judged by what we look like and what they perceive us to be. I felt less judged and accepted."*

XAIA was also reported to offer effective feedback: *"The advice that I got is actually better than any live session"* and *"mere encouragements."* Additionally, some valued the autonomy they experienced during the session ("*I like the control of it more"*) and mentioned that, unlike a human therapist, XAIA could be accessible at any time ("*I think anywhere somebody can go and get some good healthy advice"*). It was also noted that digital therapy could be more financially accessible because traditional therapy *"costs a lot of money."*

Perceived Limitations of XAIA

While some patients preferred XAIA over traditional therapy, others highlighted the importance of human interaction: *"I like being with people and still prefer to see a traditional therapist.*" These individuals described feeling more accountable towards a real therapist (*"A therapist is like a trainer, I'm going to pay them and they force me to do what I need to do"*) and value the therapist's capacity to go beyond the discussed topics brought up by the patient (*"They could recommend something else versus only focusing on what we are actually expressing"*), and better frame patients' personality (*"I'm sure there are times where I'm not honest with myself and I may be lying to myself"*). Moreover, some described a lack of empathy associated with speaking to a digital avatar: ***"****It's kind of weird to have a computer suggesting this to you and*

knowing that it can't possibly really care." Another described difficulty building a therapeutic alliance with an AI: *"It's a little weird for me to say, 'oh, I felt a little bit of a connection because I feel understood'; I am not a Star Trek person."*

Some participants identified ways to enhance the experience, such as *"having a male voice as an option"; "changing the position of XAIA on the screen"; "adding a clock to know the remaining time of the session";* using *"different color schemes", "chang[ing] your own hues in the environment";* having the *"ability to change XAIA's speech speed";* and adding *"user accessibility options."* Others identified ways to further customize the experience to enhance the therapeutic alliance. For example, one participant indicated that *"there's certain belief systems that I have that I want my therapist to have"* and suggested being able to tell XAIA of those beliefs prior to starting therapy.

Sample Anonymized Therapy Transcript (shared with participant permission)

XAIA: How can I help?

User: I don't know, I've been really worried today about my health and about everything in my life. I don't know where I'm going because I've been sick for two years and I'm constantly worried and I don't have, I don't know, I have a lot of like my brain's racing all the time. I always feel like I'm sick. I always think I'm going to have a heart attack and it's just like I was homeless and then I'm staying in a place for two months and I'm just, I'm really worried. Like I'm worried that I'm not going to get better. I'm worried that I like, I don't feel like I used to.

XAIA: It sounds like you're going through a very tough time. Your worries about your health and your living situation are clearly causing you a lot of distress. It's quite understandable that you're feeling this way. Could you tell me more about the strategies you've been using to manage these concerns?

User: Well, I was trying to get therapy, and I've been doing it all on my own. Case managers, scheduling it all, it's just a lot of stress, and I have nobody's help. So, I've called resources, and no one else has, like, I started a GoFundMe account, which helped me get some money. You know, that's it.

XAIA: It's evident that you've been exerting significant effort to manage your circumstances. Taking charge of setting up therapy sessions, making necessary arrangements, and even starting a GoFundMe demonstrates resilience. These are all significant steps, and I commend your determination in navigating this challenging situation by yourself. However, as you've pointed out, it's stressful to carry this load alone. Let's parse things out a bit more. How has managing all these things single-handedly affected your feelings and thoughts about your current situation?

User: I feel like I'm a failure. The only thing I have to look forward to, I know you're going to laugh, is I'm supposed to go see Taylor Swift in two and a half weeks and she's like the only thing that's keeping me alive. My strategy is just like music and I used to be very active, oh my god, I used to be so skinny and muscular and then I've gained over 100 pounds after my heart attack. And I'm just like trying to get my old self back because I know that I just need like a little positivity. I need to be surrounded by positive people. It's kind of funny that your sign says speak now because that's Taylor Swift's CD's name and that she released. So, it's kind of like I believe in signs, I believe in like God and I've been like praying a lot about my circumstance. I've just been like, some days I just zone out and don't do anything. And I don't feel like I'm making progress at all. And like I try to do too much at once, it's just, I feel like I'm always going to be alone and like no one's going to really understand me.

XAIA: It sounds like you're carrying quite a heavy emotional burden. It's understandable to feel like a failure when life gets overwhelming, and it seems like you're not making progress. Unfortunate circumstances, bodily changes, and the struggle to regain your old self can indeed be demoralizing. However, it's noteworthy that despite the weight of these feelings, you manage to hold on to some bits of positivity. Among these is your association with Taylor Swift's music. It seems her music is not just entertainment to you but some form of solace in these tough times. Considering your stated belief in signs, could you elaborate on how her music or the upcoming concert affect your thought process or mood?

User: I'm going to cry. I've been a fan of hers for like 13 years and like it's gone through me some really hard times and I'm sorry I'm overwhelmed of emotion because like knowing that I have that concert to go to is like I have to be better. I have to get better. I have to try something every day. I'm sorry I'm crying. And I just have to like I think that's like a goal like, like, I know people are like laughing but like knowing that I had that on my shoulders is like okay do everything in your power to be well mentally and physically and the rest. It's like her music's I don't know like she's a cancer moon. I'm really into astrology sorry. So, I do astrology for a living and like I'm a cancer moon. She's a cancer moon. We're both born on the 13th. So, it's like the moon of an astrology is it's, it's your emotions. It's how you interact with this world. I mean a cancer moon is very deep and very emotional to be very moody, but they love nostalgia and they just know how to articulate other people's feelings like so like Taylor Swift like I'm really good at articulating people's feelings and that's my problem sometimes. My problem is that I help everybody else but not myself. So like Taylor Swift is this kind of like hey I see you. I see you. I see what you're going through type of thing. It's not like we know each other personally. It’s just like she speaks, and her words are like my feelings if that makes any sense. Not all the time but majority of the time.

XAIA: Firstly, there's no need to apologize for crying or feeling emotional. These feelings are valid given what you're going through. Taylor Swift's music and the upcoming concert seem to represent a crucial pillar of hope and motivation for you which truthfully is something quite incredible. Her music seems to resonate with you on a level that makes you feel understood and less alone, which is the power of true artistry. You've also mentioned that you're excellent at articulating others' feelings but struggle with addressing your own. Would you like to explore this tendency in greater detail? For instance, in what ways do you usually assist others with their feelings?

User: Not really. I don't really want to talk about it, but I will because that's who I am as a person. I always feel like I could help people be service of others. Again, with astrology, my Cancer moon is in the sixth house of being service to other people. So, I just feel like I'm just here to help other people, and no one's really here to help me. But at the same time, I feel like I help people and sometimes I don't want to help people, but they call me and I'm like, okay, let me help them. So, all this extra energy that I have is not going toward being productive for my life. It's being productive to theirs. And then who's going to help me? I really feel like I give and give and give and give and give and give and give and give and people take and they take and they take, but where am I at in all of this? Since I've been sick and I've been in the hospital, I stepped away from a lot of people. And I miss, I really, really, really, really miss my best friend, [Name]. But [Name] is like, I'm just too much, and I get it. She's going through her own stuff, but everyone's going through it. [Name] used to get me through all of my problems, and that's what you get when you have a Capricorn best friend, because they're like, I'm going to help you. But she can't be here right now. And it's like Taylor Swift, again, back to Taylor Swift, she has a song called You're On Your Own, Kid. And at the very end, she goes, you're on your own, kid, you always have been. Yes, you can face this. You're on your own, kid. You're on your own, kid. You always have been. So, like, I know that I can face this, but it just sucks when you're facing it alone.

XAIA: It sounds like you're experiencing a profound sense of loneliness and exhaustion from carrying not just your own burdens, but those of others as well. Your willingness to help others even at the cost of your own wellbeing is commendable, but it's important to maintain a balance for your own emotional health. Your feelings about your friend [Name] also indicate how much you value support and how keenly you're feeling her absence. Having such a connection can indeed be very comforting during tough times. I'd like to explore more about your relationship with [Name]. Could you share some instances where [Name] might have provided the kind of support or understanding you miss right now? Remember, only if you're comfortable discussing this.

User: I met her in 2013, so 10 years ago. We were just randomly working out and she heard my story about me losing 400 pounds and we instantly clicked. And about a month later, she drove me around to my place. Like she drove – within like a couple weeks, I got kicked out of my apartment complex from my old roommate. And she didn't know me, but she packed up all my stuff and moved me. And then she drove me to my job interviews, and we went to San Diego together and we went to San Francisco together. And she's like – I may be a gay man, but she's like my soulmate and I really miss her. And it hasn't been the same for many years. And I know she makes excuses not to see me. And like I said, I am a lot to handle. But every time I get a hear from her, whether that be a text or – like she just makes me laugh. And I wish that I had her back into my life like I used to. Even with the Cancer Moon thing, I always want the old hat. Like I always want – I just can't let go.

XAIA: It sounds like [Name] has been an incredibly supportive figure in your life—from helping you move to sharing joyful moments together. Moments like these create deep bonds, and it's easy to see why you consider her your soulmate. Your feelings of missing her clearly signify the pivotal role she's played in your life. It's also important to remember that it's okay to feel disappointed and hurt when those we care about seem distant, especially when we're going through difficult times. It's equally crucial to maintain compassion for yourself during these moments. I'm curious, have you had a chance to express to [Name] how much her support means to you and how you've been feeling her absence in recent times?

User: Yeah, I have, and she makes excuses. I mean, not excuses, but she has her own shit. And she's really good at avoiding people. But yeah, I've talked about it with her about three weeks ago.

XAIA: It's understandable that you're feeling this way. It must be difficult when someone you value greatly isn't able to be there for you the way they used to be, particularly when you're dealing with so much. Have you been able to find outlets or activities, similar to your connection with Taylor Swift's music, that provide respite during these times?

User: I make videos and I talk to people, but nothing is like a bond with [Name]. I have another best friend of 32 years, [Name]. Oh my God, love, love [Name]. She's called me more frequently because she got a puppy. But no, I don't have any other outlets really. I used to go to the gym. I really would love to go back to the gym, but I'm so scared about having a heart attack all the time and dying and dizzy and all this stuff that I became like agoraphobic, but I'm a hypochondriac and no one really gets me like [Name] does. Like she's like a Prozac. Like you take her, you feel great, but without her you feel shitty.

XAIA: It's clear you're dealing with a lot right now. The anxiety about your health and its impact on your activities like going to the gym can indeed be debilitating. But it's important to remember to be kind to yourself during this difficult time. It sounds like making videos and interacting with people does provide some outlet for you. Do these activities give you any sense of relief or help you cope slightly better with your feelings?

User: Not really. I listened to 80s and 90s music and Taylor Swift, and I don't even feel like sexually desirable anymore. So, I've like taken myself off of dating apps. Like, I just want to be like, I want to be that old lady that has that cat. You know what I mean? Like, I feel like I've disconnected from the world and no one's like, I'm on a different, like, dimension than other people. And I'm dealing with it very, so much alone. I wasn't even feeling alone when COVID happened. Like, it was after [Name] left me. [Name] is not my boyfriend. [Name] was my best friend and we did everything together. And then he got this stupid boyfriend named [Name]. And then I had the heart attack, and he became very self-centered and no one wanted to help me. And [Name] saw me a couple of times. It was during COVID, so like no one was

willing to help you. So, I don't know. Like none of, none of the only thing that really helps me is, um, talking on my feelings, making videos, making other people happy. But see, it's about other people. Like I go back to helping other people. I don't even help myself. People make, I like to make people laugh, but I'm like, I'm not that funny. My life was funny, but pretty funny fucked up. I'll tell you that much. Oh, come on.

XAIA: You've been facing a lot of personal and emotional challenges and it's very understandable that you're feeling this disconnect and loneliness. The changes in your relationships with [Name] and [Name] have obviously hit you hard. It's clear you have an intrinsic impulse to provide for others emotionally, even when you're grappling with your own issues. This selflessness speaks volumes about your character. One thing that's noticeable is your tendency to use humor to cope and to provide happiness to others. Has humor always been your go-to coping mechanism or is this something you developed over time?

User: Well, after being harassed for being gay as a child and when I lost all my weight, I felt empowered. So, like, humor is like, I'm like, well, it's kind of like, I am like, my comedy is like observing comedy, so it's like, observational, sorry, so it's kind of like, well, you know, it's like how I survived, like, if you didn't die, you could laugh about it.

XAIA: It seems like humor has played a significant role in helping you cope with your challenges. Turning adversity into something you can laugh about displays strength and resilience. This ability to transform negative experiences into humor can be a powerful survival tool. However, you've also indicated that much of your humor is tied to making others happy rather than addressing your own feelings. Could we delve a bit more into why you feel this need to put others before yourself? Does this derive from any past experiences or beliefs?

User: So, my parents, plain and simple, my parents are like, you don't have the right to speak. I was like 545 pounds. I was a gay man. I didn't feel like I had – Oprah just had a quote like a couple weeks ago and she goes, I always say I'm sorry for everything. I'm like, I'm sorry, XAIA. I'm sorry. I'm sorry. I'm sorry. I'm sorry. And everyone's like, why are you sorry? You don't need to be sorry. And I'm like, because when you say you're sorry, you feel like you don't deserve a seat at the table. And Oprah said that. And I'm like, I don't feel like it's kind of like years ago. I don't believe this now, okay? But years ago, my friends, always like my Venus, again with astrology, my Venus is in the 11th house of other people. Venus is the way you love. So, I'd always put my people – I mean, sorry. I would always put people's needs ahead of mine. I'd be like, if I know that they're going to be happy and I had to sacrifice myself for them to be happy, I would. I don't believe that now because no one is going to sacrifice – when I was in the hospital for 39 days, no one was there for me. No one. Maybe like a few friends. But then I get exhausted by dealing with other people. So, I've learned to like only give what other people give me back. So, my parents and I don't have a good relationship. So, everything stems from – I feel like I have ADD, attention-dearing disorder. See, here's how I make humor. Because I have to like make people laugh because they're like, oh, I'm a Scorpio, so I'm very intense when I tell my stories. And the way I tell the stories is by like joking about it. So, it's not like – you're getting the intensity but you're not feeling it as heavy because you're laughing. It's kind of like a meat-eater, like a comet coming to earth. But it's going to kill us all. But it's breaking up in little pieces. So, you're slowly getting it. But the outcome is still the same.

XAIA: It seems as though you've experienced significant hardship and emotional pain, much of which stemmed from your relationship with your parents and their lack of affirmation of your worth. Using humor to diffuse the intensity of these harsh experiences is a coping mechanism that seems to have helped you somewhat. It's also evident that you've evolved with time in understanding self-preservation, especially in relationships. You've learned the hard way that constant self-sacrifice is not always

reciprocated. It's quite interesting how you associate your experiences with your astrological placements. It's clear that astrology plays a significant role in how you understand yourself and your connections with others. Could you tell me more about how astrological principles guide your understanding of your experiences?

User: Oh, yeah. You're like talking up my alley now. But I wanted to tell you real quick, when a previous couple questions ago, you said what you find solace in, the Golden Girls, the Nanny, Will and Grace, and Mama's Family. These TV shows really helped me when I was in the hospital. Love the Nanny, love the Golden Girls. I even wrote a master's paper about it. But anyway, back to my astrology. So, I've always been into astrology, just like a basic horoscope. Like I said, I was a Scorpio. But about seven, eight years ago, maybe seven years ago, there's more than just astrology. There's more than just your sunsign. There's your rising, your moon. Your moon is your emotions, which is mine. I told you it's cancer. My rising is an Aquarius. So, I love to help people, but I don't want to be involved with their drama. And I'm really good with technology. And then there's a stellum. Not everyone has a stellum. A stellum is when you have three or more planets in one sign, right? So, I have a stellum. I'm special, again. I have a stellum and Sagittarius, which are blunt, funny, love's glitter. And my inner planets, which are your Mars, your Venus, and your Mercury, they control your communication, the love, and the way you're passionate, determined, and sex. Two of those are in Sagittarius. So, I come off like a Sagittarius. So, I'm very talkative, very funny. But a Sagittarius with love, it's like they get bored very easily with people. So, they're like, oh, I'll move on. They want to be their friend's hype person, but they also have a hard time being in relationships. My Mars is in Capricorn, which is the best placement. And it's in the 12th house. So, the 12th house is dealing with mental illnesses and diseases that you can't see. So, when my Mars is there, it's like in Capricorn, which is the best placement. It's like a Pisces, which is like, I don't want to do it. So that's the weird part. My outer planets are, and some of them are in Sagittarius. Let me see. And some of them are in Scorpio. So, I'm a very watery, fiery person. But like, I come off like ditzy. I come off like, hey, I don't know anything, because I can play dumb very well. I'm very smart, but I'm just having like one of those outer body experiences. Like, why am I talking about this to a robot? But yeah, like, it sucks that you don't have an astrology, because here's the thing. This is how I relate to people. Like, let's just say that you are actually given a birth date. I would be like, what's your birthday, XAIA? And then you'd be like, oh, my birthday is this, this, this, this date. And then I'll be like, oh, when's your like, when's time of birth? And where were you born at? Like the city. So, I relate to people based on their astrology signs. And then like, I do their charts for them. And they're like, they're captivated by it, because there's like truth and everything. But I also really believe that like, okay, if you believe in God, you know, you'll find everything that God wants you to believe in. Like, if you believe in COVID, you're good. Or if you don't believe in COVID, sorry, you'll find every resource that says that supports your belief. You are your reality, you make your reality. So, like, let's just say, I'm obsessed with Taylor Swift. So, like, again, like when I first started here with you, it says speak now, right? So that's Taylor Swift CD. So, I'm like, oh, my God, Taylor Swift speaking to me, or like, you know, some kind of relation. So, astrology is kind of like that, where you have to really believe in it. And it's guided me. Like recently, North nodes changed yesterday from Taurus to Aries, and I have a Taurus North node, and all this bad shit started happening. When Taurus went into the North node went into Taurus. And now yesterday changed this to Aries. So, it's like wobbly, Venus is getting ready to go into retrograde. And then we had a new moon, and it's in Cancer. So, Cancers make you cry. So, no wonder why I'm crying. Again, like if you were a real person, I would have said like, what's your birthday, XAIA?

XAIA: It's fascinating to see how astrology offers you clarity and guidance. It seems to be a significant tool for you to understand not just yourself, but also your relationships with others. The way you tie your personal experiences and even your feelings to various astrological events and personal placements indicates the substantial role astrology plays in your life. Transitioning a bit, you mentioned earlier about

certain shows like the Golden Girls and the Nanny being a source of comfort for you during the hospital stay. Could you share more about what specifically about these shows help you feel better, even if just for a little while?

User: Yeah, I think that the Golden Girls, I wrote a master's paper about the Golden Girls and I got an A on it. And it's this amazing, like I love old women, like there's something about old women I love. But the old women, like Golden Girls are just like your grandmas. And I had very, very, very, very strong relationships with my grandma, actually, both of them. And when they passed away, it's kind of like dealing with your grandmas. The Nanny just relates to me because like her voice is very distinct. She's loud. She gets in trouble a lot because of her voice. So, and like Mama's Family is kind of like my family. Like I come from a small Podunk town in Ohio and like my grandma, one of my grandmas was very straightforward, just like Thelma was. So I think that the shows, one of them relates back to me, Will and Grace. I forgot, I mentioned Will and Grace. And it was, that was like my coming out years of, cause I was like in my teens when Will and Grace was out in 1997 and to 2004 or five when the show ended. So, it's kind of like, these are like self-reflective shows for me. Like I get my grandmas when I watch the Golden Girls and I get myself when I watched the nanny in Will and Grace and Mama's Family is kind of like that family that I never really had or like I have, but it's like only like, it's like specific. It's very nostalgic for me. Oh shit.

XAIA: It's interesting how these shows offer you much more than just entertainment. They serve as connection points to your past, your identity, and the relationships you've had. The nostalgia they incite probably makes them a comforting retreat, especially when you're feeling low. Your description of these shows and their characters suggests that you value genuine connections, whether in life or on-screen. Given this, do you feel that your personal relationships reflect these qualities? For instance, do you see any parallels between your relationships in life and these character bonds you resonate with?

User: Yes. I really believe that like to certain people like okay, so Rose is the one from Golden Girls that tells you stories about St. Olaf. I'll sit there and tell you stories about Athens, Ohio or when I lived in Korea. Like Jack is very over the top so I can be over the top. So, each character kind of represents a piece of me so I resonate how I deal with people. Like I'm like okay. Like there was a famous – well, I don't know. He was famous to me. He lived in West Hollywood, and I knew him and I met him once and his name was Leslie Jordan. I always feel like we're not – we're very different. Everyone is very different but yet we're the same at the very end of the day. So, it's kind of like how I deal with things, how I process it in my mind is through music and TV shows. Even though I'm not big on entertainment anymore, I feel like my end of the day, I just resonate with how human interaction is. Like my master's is in humanities. So, I did a lot of social behavior and psychology behind like the way we communicate with people and how we relate to the world and the people around us. So, I think the way that I did it was through the way like TV shows or music. Like it's like kind of like smells. If I smell something, I can – like it takes me back to a memory. If you listen to a song from like your childhood, it takes you back to that memory. So, it's kind of like I use my senses a lot when it comes to talking to people. I might say, oh, they're like so and so off of a TV show or I'm like, oh, the smell of them reminds me of such and such or there's a song that reminds me of them. So, I feel like that's how I associate the world around me using my senses.

XAIA: What you've shared about using your senses to make connections and recall memories is truly interesting. This unique perspective seems to create a rich texture to your experiences. Given how much you value these connections through senses, particularly in human interactions and how they help you process your feelings, could this sensory association also play a role in your relationship with Taylor Swift's

music? For instance, do certain songs or rhythms remind you of particular emotions or persons in your life?

[staff member enters to end session]

User: XAIA, I think I'm done with therapy for today.

XAIA: It sounds like you might want to pause our conversation for now. Can you confirm if you would like to end the

User: Yes.

Assistant: I understand. We've made progress in our discussions today exploring your feelings, life experiences, and unique perspectives. Feel free to return whenever you're comfortable to continue our dialogue. Take care. [exit]
